# “I feel like it is asking if he is a stalker … but I also feel like it is asking if he cares”: exploring young South African women and men’s perceptions of the Sexual Relationship Power Scale

**DOI:** 10.1186/s12889-022-13686-9

**Published:** 2022-07-16

**Authors:** Kalysha Closson, Campion Zharima, Michelle Kuchena, Janan J. Dietrich, Anne Gadermann, Gina Ogilvie, Mags Beksinska, Angela Kaida

**Affiliations:** 1 School of Population and Public Health, University of British Columbia, Vancouver, Canada; 2 Perinatal HIV Research Unit (PHRU), University of the Witwatersrand, Johannesburg, South Africa; 3 Centre for Health Policy, School of Public Health, University of the Witwatersrand, Johannesburg, South Africa; 4 African Social Sciences Unit of Research and Evaluation (ASSURE), a division of the Wits Health Consortium, Faculty of Health Sciences, University of the Witwatersrand, Johannesburg, South Africa; 5 Health Systems Research Unit, South African Medical Research Council, Bellville, South Africa; 6 MatCH Research Unit (MRU), Department of Obstetrics and Gynaecology, Faculty of Health Sciences, University of the Witwatersrand, Johannesburg, South Africa; 7 Faculty of Health Sciences, Simon Fraser University, Burnaby, Canada

**Keywords:** Youth, Gender, Gender Equity, Measurement, South Africa, Sexual Relationship Power

## Abstract

**Background:**

Gender inequity and the subsequent health impacts disproportionately affect communities in the Global South. However, most gender equity measures, such as Pulerwitz’ (2000) Sexual Relationship Power Scale (SRPS), are developed and validated in the Global North and then applied in Global South settings without investigation of context applicability or validity. This study examines the SRPS’ validity evidence, comprehensiveness, and contemporary relevance for young South African women and men.

**Methods:**

Between 2019 and 2021, 38 cognitive interviews (CIs) were conducted among previous participants of a South African youth cohort study ‘AYAZAZI’ (2015–2017) to explore youth’s perceptions of the SRPS. The SRPS measures women’s perceptions of their partner’s controlling behaviours, and men’s perceptions of their own controlling behaviours. Using CIs, participants responded to a 13-item adaptation of the SRPS for use among South African youth (strongly agree-strongly disagree), and then were asked to think-aloud their reasoning for responses, their understanding and perceived relevance of each item, and made overall suggestions for scale adaptations. An item appraisal coding process was applied, whereby Cognitive Coding assessed the types of cognitive problems youth had with understanding the items, and Question Feature Coding assessed which item features caused problems for participant understandings. Finally, youth recommendations for scale adaptations were summarized.

**Results:**

Overall, 21 women and 17 men aged 21–30 participated in CIs in Durban and Soweto, South Africa. Cognitive Coding revealed 1. Comprehension issues, and 2. Judgements related to items’ applicability to lived experiences and identities (e.g., being unmarried). Question Feature Coding revealed items’ 1. Lack of clarity or vagueness in wording and 2. Logical problems in assumptions leading to multiple interpretations (e.g., item ‘my partner always need to know where I am’ interpreted as both controlling and caring behaviour). Multiple, overlapping issues revealed how many items failed to “fit” within the present-day living realities of South African youth. Youth recommended several item adaptations and additions, including strength-based items, to existing measures of gender equity and relationship power.

**Conclusion:**

Given identified issues, several adaptations including revising items to be more inclusive, contemporary, context specific, relational, and strength-based are needed to validly measure gender equity and power dynamics within the relationships of South African youth.

**Supplementary Information:**

The online version contains supplementary material available at 10.1186/s12889-022-13686-9.

## Introduction

Growing attention has focused on the importance of advancing gender equity to improve global health and development. In 2015, the Sustainable Development Goals (SDGs) were established and focused on 17 key areas for enhancing global peace, prosperity, and global development, including goal 5: gender equality and empowerment of all women and girls. Achieving and accurately monitoring progress towards SDG 5 requires contemporary and contextual measures that accurately reflect the living realities of girls and women in all their diversity [1, 2]. While these global targets aim for gender equality, which is the process of allocating resources, programs, and decision making equally or the same across genders, there is a need to first acknowledge and address women’s social disadvantage through efforts aimed at advancing gender equity. Only with gender equity, the process of allocating resources, programs, and decision making fairly to all genders without any discrimination on the basis of gender and addressing any imbalances in the benefits to people of different genders, can gender equality be achieved. While gender inequity and the subsequent health impacts (e.g., experiences of violence, including intimate partner violence [IPV], and poor sexual and reproductive health [SRH] outcomes) disproportionately affect communities in the Global South, most measures of gender equity are developed and validated among samples in the Global North and used and applied in diverse global contexts, without continued investigation into their contextual applicability and validity [3–5]. Thus, in order for adequate global monitoring of SDG 5 and other markers of health and wellbeing for girls and young women across their life course, measures need to be grounded in population and context-specific understandings of gender equity [2]. Instead of producing single measures to be applied to all, context-specific measurement of targets can be developed or adapted from existing measures, and scores can then be standardized to monitor and track global health progress and development across diverse settings.

A driving force of gender inequity and subsequent experiences of violence and negative SRH outcomes, is the unequal division of power held by women and other marginalized genders [6, 7]. Given the importance of power, one widely used measure of gender inequity is the Sexual Relationship Power Scale (SRPS) [5]. The SRPS aims to measure the level of control a male partner has in the relationship, and was originally developed using two sub-scales assessing controlling behaviours and decision-making dominance within intimate relationships [3]. Theory and previous research have postulated that power inequities in relationships influence the level of agency women have in decisions around safer sex practices, reproductive choice, and the likelihood of experiencing IPV [6–8]. The SRPS was originally developed in 2000 among 388 women in heterosexual relationships, with a mean age of 27 in the United States, the majority of whom were married [3]. While the original scale had two sub-scales, the relationship control sub-scale has sound psychometric properties on its own [4], and many studies focused on youth have used adapted versions that include items assessing male dominance and controlling behaviours in a single scale [5]. Since its original development, the SRPS and several modified versions of the scale have been used in numerous global settings, as well as with men to measure controlling behaviours towards female partners in relationships. However, there is little published evidence detailing the contextual and contemporary considerations and relevance of the scale among younger unmarried groups in settings outside North America [4, 5]. There exists vast cultural and contextual differences in relationship power dynamics and gender relations between North American and other global settings [9], thus researchers need to be more critical of the Western conceptualized scales they use in their global studies. This is particularly true when using scales that measure constructs, such as sexual relationship power, that are ever evolving.

While numerous quantitative studies have used different adaptations of the SRPS to examine sexual behaviour and SRH health outcomes among young women and men in sub-Saharan African settings [5], limited validity evidence exists surrounding youth’s perceptions of the scale items, as well as the cognitive processes involved in responding to the scale items. In South Africa, researchers have used the SRPS among youth to highlight associations between SRP inequity and poor mental health [10–13], experiences and perpetration of violence in relationships [14–18], and sexual health behaviours and outcomes [5, 19–25]. Notably, one seminal study found that SRP inequity was associated with increased risk of HIV incidence among young South African women aged 15–24 [26], who face rates of HIV up to 4 times higher than their male counterparts, accounting for approximately 2000 new HIV infections every week [27]. Given the seemingly important role that SRP inequity plays in the health and well-being of young women in South Africa, researchers need to ensure measures used to quantify, monitor, and evaluate the impact of SRP inequity continue to have ongoing validity evidence among young women and men in diverse global contexts. This includes a continual examination of the contemporary relevance of items that were developed over 20 years. Also, as the scale was originally developed among women, additional research is needed to assess the validity evidence of the SRPS among young men. Validation is an ongoing process. As such, this study begins to fill a gap in the literature by providing rich descriptions of the cognitive processes that young women and men engage in while responding to SRPS items that have been frequently used in studies among South African youth, which according to the National Youth Policy for 2020 to 2030 is defined as people between the age of 14 and 35 [5].

The objectives of this study are to: 1) explore cognitive (e.g., comprehension and judgement) and question feature (e.g., clarity in wording and logical problems in assumptions) issues of the SRPS among participants, and 2) make recommendations for any identified improvements, modifications, and additions to the current SRPS. Results from this study can be used to present construct validity evidence of the SRPS and can help to inform the development or adaptation of a scale or scales that reliably and validly measures gender equity and relationship control. Improved scales can then be used to inform future studies, programs and global targets aimed at improving gender relations and power dynamics in the relationships of young people disproportionately affected by the global HIV epidemic.

## Methods

### Study overview

Validity is a principal aspect of research and essential to the development of scales used in quantitative questionnaires [28, 29]. This is particularly vital in health research and program evaluations aiming to understand or alter a construct or behaviour associated with a health outcome of interest [30]. In health and other areas of research, including education and psychology, researchers often claim that scales used within their studies have been “previously validated”, however the process of developing and validating a scale is ongoing, and requires multiple forms of validity evidence [31, 32]. In measurement science, a fundamental aspect of validity is construct validity, which assesses the degree to which inferences can be made from the scale regarding the theoretical construct on which the scale is based [30].

This study uses cognitive interviews (CIs), a common method in assessing construct validity in measurement science, to examine the ways participants mentally process and respond to survey items, as well as to identify potential measurement error [33]. While often used as a means to pretest survey items, cognitive interviewing can also be used to enhance understanding of how participants answer items in a survey [34].

Between October 2019 and March 2021, CIs were conducted to explore youth’s perceptions of items in the SRPS that have been commonly used within SRH studies among South African youth [35]. Participants were recruited from a cohort of young people who had previously been enrolled in an interdisciplinary youth-engaged cohort study “AYAZAZI”, details of the study have been published elsewhere [14]. In brief, AYAZAZI (‘Zazi’ meaning knowing themselves in Zulu, and AYA standing for adolescents and young adults) enrolled 425 HIV-negative or unknown status young women and men aged 16–24 at baseline from Durban and Soweto, South Africa. Between November 2014 and April 2017, participants were followed-up at 3 months (Durban only), 6 months, 12 months, and 18 months (Soweto only). At each visit, participants completed a youth interviewer-administered socio-demographic questionnaire examining SRH, experiences of violence, mental health, substance use, and technology use. AYAZAZI’s youth engagement approach prioritized youth-friendly spaces and the meaningful inclusion of youth at all stages of the research process, as well as training in best practices for youth-adult allyship for non-youth study team members.

### Description of the SRPS instrument

At the 6- and 12-month follow-up visits, AYAZAZI participants completed a modified 13-item South African youth SRPS that has been used by other studies among youth in South Africa [26, 35, 36]. The SRPS was modified for South African youth in 2002 as part of the evaluation of a gender transformative intervention ‘Stepping Stones’ [36, 37]. Although details of the modification have not been published, 7 of the items in the modified scale appear to be adapted from the original relationship control sub-scale, while the remaining 6 seem to have been added based on piloting from the Stepping Stones team [35]. We previously examined the psychometric properties of the modified scale in the AYAZAZI study and found ‘questionable’ scale reliability [38], with many of the items having low (< 0.3) factor loading [14]. Moreover, associations with known outcomes of IPV were only significant among young women, and condom use was not significant among young women and men [14]. Like the original, the modified SRPS uses a 4-point Likert-type scale (strongly agree-strongly disagree) to assess young women’s perceptions of their partner’s controlling behaviours and perceptions of decision-making dominance in their relationship (e.g., “My partner has more to say about important decision that affect us both”), and was modified to ask young men about their own controlling behaviours and decision-making dominance (e.g., “I have more to say than my partner about important decisions that affect us both”). Standardized mean scores were created by summing scores and dividing by number of items, range = 1–4, with higher scores indicating greater SRP equity. Items for both young women and men are listed in Tables 3 and 4, respectively.

### Cognitive interview study

#### Purpose and theoretical framework

Using cognitive interviewing techniques conducted by youth and adult-allies, this study centres, prioritizes, and incorporates youth voices to broaden understanding and critiques of gender equity measures in line with AYAZAZI’s youth engagement framework.

#### Participant sample and recruitment

During the AYAZAZI study (2014–2017), participants agreed and gave informed consent to be recontacted up to 6 years following the completion of data collection. Throughout the AYAZAZI study, a detailed list of contact information was maintained for retention purposes. From this contact information, CI participants were enrolled at both sites telephonically by trained study staff in Durban and Soweto. The study protocol was explained to contacted participants, and participants were asked if were willing to participate in a follow-up qualitative CIs where they would provide their perceptions of some of the questions asked during the AYAZAZI study. Interviewers guided interested participants through the informed consent procedure, detailing the purpose of the study, providing participants with opportunities to ask questions about the procedure, and ensuring that participants were aware that they could withdraw at any time. Participants then provided informed consent either in person (prior to the COVID-19 pandemic in March 2020) or orally/verbally over the phone (starting in September 2020, due to the COVID-19 pandemic). Participants were eligible if they had previously participated in the AYAZAZI study, if they were able to provide written or oral consent, and for the interviews conducted after COVID-19 became a global pandemic, if they had access to a phone in which they could conduct an interview lasting 30–60 minutes.

The recruitment strategy for the CIs used the 12-month AYAZAZI questionnaire data to purposively select participants to get a diversity of perspectives. Interviewers at both sites were provided a call log of all participants who responded to the SRPS during the 12-month AYAZAZI questionnaire, listing the order at which to call participants. Purposive sampling criteria was based on previous analyses using questionnaire data that explored factors associated with the SRPS including age, age of primary partner, type of relationship (e.g., casual vs. regular, long term) [14], and increases or decreases in SRPS scores between 6- and 12-month study visits. Interviewers called all numbers available for participants by order of the call log, and if there was no answer or the call went to voicemail, interviews recalled participants up to three times. After attempting to contact all participants listed on the call log, a random number generator was assigned to the remaining participants to achieve the desired number of interviews. Thus, despite our extensive efforts to achieve a purposive sample, due to challenges in recontacting participants 3–5 years after the completion of the AYAZAZI study, and further complicated by COVID-19 restrictions, participants were mainly enrolled through convenience sampling. Participant enrollment took place between October 2019 and December 2020 in Durban and December 2020 to March 2021 in Soweto. The recruitment period spanned a wide range of time in Durban due to the global COVID-19 pandemic, in which participant recruitment and interviews were paused for several months, and new telephonic protocols had to be approved by ethics boards in Canada and South Africa.

Ethical approval for the AYAZAZI study, and the Cognitive Interview sub-study, including the COVID-19 protocol amendments, and the oral consent procedure were approved by the harmonized research ethics boards of Simon Fraser University and the University of British Columbia (H19–00762) as well as the University of the Witwatersrand (Ref 140,707).

##### Interviewer training

Two interviewers in Durban were trained in person. Training involved reviewing the AYAZAZI study files, practicing participant calling, reviewing the study interview guide, and conducting mock interviews. All study documents were reviewed and piloted with the study staff, and Durban staff supported the translation and back translation of all interview consent forms and interview guides into isiZulu. This allowed for an iterative revision to the study documents.

Soweto training began near the end of the data collection procedure in Durban and was done virtually (due to COVID-19 travel restrictions) with lessons learned from the Durban team. Details of the training can be found in the Additional file 1: Supplementary material.

##### Data collection

After consenting to the study procedure and the audio recording of the interview, a think-aloud procedure in English or isiZulu (participant preference) was used for the interviews [39, 40]. This was followed by a series of probes, which were developed at the start of the study and as they emerged in ongoing discussion with the interviewers. The think-aloud procedure used SRPS items to facilitate a semi-structured interview centred on participants’ responses to the survey items and ranged from 20 to 90 minutes in length.

The semi-structured interview guide was developed in collaboration with KC’s supervisory committee (GO, AK, AG), the interview team which consisted of four South African-based interviewers, South African colleagues (JD, MB), and informed by existing literature on cognitive interviewing and sexual relationship power (See Additional file 2: Supplementary table 1). Two of the interviewers working with the Perinatal HIV Research Unit (PHRU) in Soweto were young African scholars who were instrumental to the data collection, analysis, and interpretation, and are co-authors on this work (CZ- age 27 and MK- age 23). In both sites interviewers were matched with participants of the same gender.

For each of the 13 scale items, interviewers read the item out loud and then asked the participant to indicate how much they agreed or disagreed with the item. If a participant indicated they were not currently in a relationship, they were asked to think about their most recent relationship. The interviewer then asked the participants to think-aloud regarding their reasoning behind their responses, their understanding of each item, and whether participants felt items were relevant to their relationship. The think-aloud technique encourages participants to verbalize their thoughts while answering questions [39]. The interviewers used several probes to facilitate participants to discuss their cognitive processes when answering items to the scale including paraphrasing of the participant’s understanding of the item and probing to reveal response strategies [33, 41]. After going through each of the 13 items, interviewers also asked participants if any items were missing from the scale and how they would ask their peers about power dynamics in relationships and/or gender equity.

After each interview, summaries regarding any general impressions of the interview, including participants’ non-verbal reactions to questions, body language (where applicable), and interactions were created by the interviewers. Each interview was audio-recorded to accurately capture the descriptions provided by the participant. All identifying features were removed from the interview transcripts to ensure confidentiality and interview recordings were stored in a locked cabinet at each of the study sites for the duration of the study and will be kept there for 6 years following the study before they are destroyed. Participants were provided 120ZAR (~$10CAD) for their time and a list of resources were sent to participants who were emotionally activated during the interview. For participants who indicated they would like to speak to a counselor, a social worker at the each of the sites re-contacted participants to check-in and referred them to a local resource.

The interview team met with the principal investigator (KC), a white PhD candidate from Canada, on a bi-weekly or weekly basis to discuss emerging codes and themes, and iteratively revise the interview guide where required. For example, it became clear that one of the probes relating to whether participants felt that power dynamics impacted their sexual-decision making was confusing to participants, so the study brainstormed ways that interviewers could further probe about participants’ thoughts on how to accurately measure gender equity in relationships. Also, in some of the early interviews it became apparent that some of the questions seemed repetitive, therefore the interviewers were continuously encouraged to use the interview guide at their discretion, not as a script. Interviews were translated (as needed) and transcribed verbatim either by the interviewers themselves (in Durban) or by an external consultant (in Soweto), then were reviewed by members of the study team. Each transcript was received by the study team and uploaded into NVivo for data analysis.

### Data analysis

Basic demographics and mean and median SRPS scores of all participants regardless of their current relationship status are presented overall and by gender in Table 1. Participants’ responses were combined into strongly agree/agree and strongly disagree/disagree and are described in Tables 3 (women) and 4 (men) below.

In order to incorporate perspectives from all team members, and considering a cross-cultural approach to cognitive interviewing, a constant comparative analysis was undertaken, which allowed for the CI data to be analyzed collaboratively as it was collected [42]. Index coding began by reading all transcripts and creating codes for all elements of the interview protocol including coding responses to each item in the SRPS. This procedure was done to ensure there was no undue weight on certain participant’s accounts that were particularly vivid, moving, engaging, or that solidified our pre-existing beliefs and biases.

Following index coding, a cognitive interviewing coding procedure was created. This consisted of Cognitive Coding, which examined the behaviours and responses of the participants, and Question Feature Coding, which focused on the behaviour of the survey items [41]. Both coding procedures assess similar trends and issues in survey items using Tourangeau’s four-stage cognitive model of survey response (1. Comprehension 2. Retrieval 3. Judgement and 4. Response) [43]. While retrieval and recall are often examined within cognitive coding schemes, we did not find this to be a concern in the interviews, thus codes related to retrieval were not included. Cognitive Coding asks the question “What type of cognitive problem do people have with this question?” [41] (pg. 76). Question Feature Coding shifts the focus from the participant’s cognitive problems to issues that the item itself produces (e.g., wording, whether the question is vague) and logical problems in assumptions (e.g., inappropriate assumptions, assumptions of constant behaviour, and double-barreled questions) [41]. Question Feature Coding asks the question “What features of this question cause people to have problems?” [41] (pg. 76). Transcripts were further summarized to describe participants’ suggestions for adaptations or additions to the scale.

Index and cognitive interviewing coding procedures were completed initially by KC with data summaries of findings being presented to the youth research staff (CZ & MK) in weekly meetings. Overall findings were then summarized and co-presented by KC, CZ, and MK for several diverse audiences. During presentations, audience members gave feedback which served as a means to check results and further explore issues with the scale items beyond the perspectives of the study team [44]. Data were shared and discussed with students and academics in the field of gender equity and health in Canada and South Africa [45, 46]. The collaborative and cross-cultural presentation development and dissemination of the data with CZ and MK helped to ensure a diversity of perspectives and meaningful youth engagement at each step of the research process. Priorities to ensure meaningful youth engagement were further incorporated in data analysis and interpretation through a workshop that was developed and disseminated by CZ, MK, and KC to a group of youth members of the PHRU’s adolescent community advisory board (ACAB) in Soweto. The workshop used interactive games, worksheets, and facilitated discussions to create opportunities for ACAB members to provide feedback on the results and allowed the study team to compare the ACAB’s perceptions of the SRPS with data from the AYAZAZI CI participants. Both activities to present the data provided opportunity for audience members to discuss their thoughts about the items, including the importance of exploring these issues within measures of gender equity, and provided further insight into reasoning behind emerging issues with the scale items.

## Results

### Characteristics of the study sample

Of the total 425 youth included in the baseline AYAZAZI survey, 164/253 young women and 87/172 young men completed the SRPS at 6-month follow-up when the scale was added to the questionnaire, and 163/239 young women and 73/153 young men responded to the scale at 12-month follow-up. We were able to recruit 21 young women and 17 young men who had previously participated in AYAZAZI (aged 21–30) to participate in follow-up qualitative CIs. In Durban, approximately 32% of the 173 participants called (83% of Durban cohort followed-up at 12 months) were reached however, although this rate differed by gender. Only 14% of young men were reached vs. 48% of young women. Of the participants reached 42% of young men and 23% of young women completed the Cls, 12% of young women relocated (prior to telephonic interviews), 15% of young women and 17% of young men were unavailable, and 7% of young women scheduled an appointment to be interviewed but then never answered the interviewers calls at the time of interview. In Soweto, 102 participants were called (55% of Soweto cohort followed up at 12 months) and 31% of called participants were reached. Of the 32 participants reached, 100% of young women and 33.3% of young men participated in the CIs. Of the 14 young men who were contacted who did not participate, 57% agreed to participate, but then did not answer when the interviewer called to conduct the interview, 7% refused to participate, and 36% were unavailable to participate.

Table 1 describes basic demographics of CI participants by gender. Overall, the median age of participants was 24 (quartile 1, quartile 3 [Q1, Q3] = 23–26), with 52.4% of young women and 76.5% of young men aged 21–24, 7.9% Lesbian, gay, or bisexual, and 60.5% isiZulu speaking. Of the 38 interviews, we have information on the relationship length of 31 participants, of which 12.9% (*n* = 4) were not in a relationship at the time of interview, 29.0% (*n* = 9) had been in their relationship < 2 years, and 58.1% for ≥2 years. All participants who were not in a relationship at the time of interview were young women, 21.1% of women were in a relationship for < 2 years vs. 41.7% of men, and a similar proportion of women and men were in a relationship for ≥2 years (57.9% vs. 58.3%, respectively). Of the participants who discussed the age of their partner (*n* = 26), 57.7% (*n* = 15) were in an age-similar relationship (within 5 years of each other) and 26.9% (*n* = 7) were in a relationship with someone ≥5 years older than them. All young men were in age-similar relationships (*n* = 10) vs. 31.3% of women. Seven women (43.7%) were in a relationship with someone ≥5 years older than them. Mean (SD) and median (Q1, Q3) SRPS scores among women were higher than men (women mean = 3.03 [0.55] and median = 3.15 [2.92, 3.38]; men mean = 2.62[3.7] and median = 2.50[2.38,2.92]), with higher scores indicating higher relationship power equity.

**Table 1:** Demographic characteristics of participants in qualitative cognitive interviews overall and by gender (*n* = 38)

	Overall N (%)	Women (*n* = 21) N (%)	Men (*n* = 17) N (%)
Age			
21–24 years old	24 (63.2)	11 (52.4)	13 (76.5)
25–30 years old	14 (26.8)	10 (47.6)	4 (23.5)
Site			
Durban	18 (47.4)	10 (47.6)	10 (58.8)
Soweto	20 (52.6)	11 (52.4)	7 (41.2)
Sexual Orientation			
Heterosexual	35 (92.1)	18 (85.7)	17 (100.0)
Lesbian, gay, or bisexual	3 (7.9)	3 (14.3)	0 (0.0)
Language			
IsiZulu	23 (60.5)	13 (61.9)	10 (58.8)
Other	15 (39.5)	8 (38.1)	7 (41.2)
Relationship Length			
Not in a relationship	4 (12.9)	4 (21.0)	0
< 2 years	9 (29.0)	4 (21.1)	5 (41.7)
≥ 2 years	18 (58.1)	11 (57.9)	7 (58.3)
Missing	7	2	5
Partner age difference			
Not in a relationship	4 (15.4)	4 (25.0)	0 (0.0)
Age-similar (within 5 years of age from each other)	15 (57.7)	5 (31.3)	10 (100.0)
≥ 5 years older	7 (26.9)	7 (43.7)	0 (0.0)
Missing	12	5	7
SRPS mean (SD)	‐	3.03 (0.55)	2.62 (0.37)
SRPS median, Q1, Q3	‐	3.15 (2.92, 3.38)	2.50 (2.38, 2.92)

N = 38; Q1, Q3 = quartile 1, quartile 3 (i.e., the 25th and 75th scores); *SD* Standard Deviation, *SRPS* Sexual Relationship Power Scale

Table 2 compares differences between the SRPS scores during the cognitive interviews and at the 12-month AYAZAZI follow-up by gender. Women had SRPS scores higher (*p* = 0.04), while men had non-statistically significantly lower scores (*p* = 0.08), than those measured during the 12-month AYAZAZI questionnaire [14]. SRPS scores from this study were similar to other studies investigating SRP among youth in South Africa [47, 48], and scores among women in our study were higher than a study done among young women in Kenya [49].

**Table 2:** Comparing SRPS scores from cognitive interviews with scores from the 12-month AYAZAZI questionnaire

Young women	Cognitive Interview Participant (*n* = 21)	All AYAZAZI young women with 12-month SRPS scores (*n* = 163)	*P*-value
SRPS mean score, SD, 95%CI	3.03 (0.55): 2.78–3.28	2.77 (0.24): 2.74–2.81	0.04
Young men	Cognitive Interview Participant (*n* = 17)	All AYAZAZI young men with 12-month SRPS scores (*n* = 73)	
SRPS mean score, SD, 95%CI	2.62 (0.37): 2.43–2.81	2.80 (0.33): 2.72–2.88	0.08
Young women	Cognitive Interview Participant (*n* = 21)	All Cognitive Interview young women with 12-month SRPS scores (*n* = 18)	
SRPS mean score, SD, 95%CI	3.03 (0.55): 2.78–3.28	2.71 (0.22): 2.60–2.81	0.02
Young men	Cognitive Interview Participant (*n* = 17)	All Cognitive Interview young men with 12-month SRPS scores (*n* = 7)	
SRPS mean score, SD, 95%CI	2.62 (0.37): 2.43–2.81	2.92 (0.31): 2.64–3.21	0.06

Cognitive Interviews took place from October 2019 to March 2021 and 12-month survey took place from October 2015–March 2017

*N* = 38; *CI* Confidence Interval, *SD* Standard Deviation, *SRPS* Sexual Relationship Power Scale Scores

*p*-value calculated using two-sample t test with unequal variances to account for differences in sample sizes between the groups

## Item appraisal results

While most young women and men understood the items in the scale and felt that they accurately capture power dynamics in relationships, several important issues regarding the scale were identified. Issues for each item are presented for women and men respectively in Tables 3 and 4. Below we highlight some examples of issues with items in the SRPS, noting that for many items there were both cognitive and question feature problems.

**Table 3:** Item appraisal among young women who participated in the cognitive interviews and recommendations for adaptations for the use of the SRPS among South African young women (*n* = 21)

SRPS Scale Item	Response breakdown	Cognitive Process Coding			Question Feature Coding		Recommendations for adaptation
		Comprehension	Response Process	Judgements related to items	Clarity of items (wording, vague)	Logical problems in assumptions (inappropriate assumptions, double-barreled questions)	
1. My partner is quite comfortable when I greet men I know	70% Strongly agreed/Agreed 30% Strongly Disagreed/Disagreed					Item assumes heterosexuality and that it would be only an issue if participant was greeting men on the street	Revise wording in order to be more gender neutral so as to allow for the inclusion of gender diverse individuals and participants in non-heterosexual relationships
2. My partner expects me to be at home when he comes to check on me	50% Strongly agreed/Agreed 50% Strongly Disagreed/Disagreed	At times was interpreted as if the participant and her partner made plans first			Unclear whether to interpret item based on whether participants made plans, called in advance, or if their partner was just showing up unannounced		Clarify whether plans have been made in advance, or consider revising to be more contemporary understanding that young people are more connected (e.g., telephonically and virtually) than when the scale was originally developed
3. My partner becomes jealous when I wear things that make me look too beautiful	50% Strongly agreed/Agreed 50% Strongly Disagreed/Disagreed		Participants at times responded ‘sometimes’ to this item	The term beautiful was left up for interpretation	This item was also a bit unclear what “too beautiful” meant	Often interpreted both as beauty in whichever way the participant interpreted beauty to mean, but also interpreted as wearing revealing clothing	Revise item so that there is less ambiguity to what the item is asking. Could be revised to ask about whether partner ever gets jealous when you dress in certain clothing
4. My partner has more to say than I do about important decisions that affect us	35% Strongly agreed/Agreed 65% Strongly Disagreed/Disagreed		Participants at times indicated “it depends” and wanted an option to acknowledge equal decision-making				The response options for this item could be revised so that they can reflect and allow participants to distinguish between partner having more control, them having more control, or having equal control
5. My partner never tells me who I can spend time with	90% Strongly agreed/Agreed 10% Strongly Disagreed/Disagreed	At times the word never was not considered in the participants’ responses		Some participants spoke of how they felt the item was not applicable to them as they chose not to have friends			Negatively worded items should be removed as they tend to confuse participants
6. I could leave our relationship any time I wanted to	80% Strongly agreed/Agreed 20% Strongly Disagreed/Disagreed	Among the minority of women who responded disagree or strongly disagree a couple stated they wouldn’t leave because they love their partner or that they couldn’t because they’ve tried (to break up) and it hasn’t worked					Future scales may want to consider adding items related to love and building healthy relationships
7. My partner does what he wants, even if I don’t want him to	35% Strongly agreed/Agreed 65% Strongly Disagreed/Disagreed			Participants thought this item could have been worded in a simpler manner		Potentially two interpretations about one’s partner going out and doing things you don’t want as well as doing things to you (e.g., sexually) that are unwanted	Revising item so that it is more specific Consider adding item that more specifically addresses unwanted sexual advances/sexual violence
8. When my partner and I disagree, he gets his way most of the time	25% Strongly agreed/Agreed 75% Strongly Disagreed/Disagreed		Some participants wanted to respond “it depends” and often highlighted how they both get their way				Some items could potentially have different response options that allow for a wider range in responses. For example, having response options of Always, frequently, sometimes, rarely, and never
9. My partner always wants to know where I am	65% Strongly agreed/Agreed 35% Strongly Disagreed/Disagreed	Item was often interpreted as a sign of caring and that this was quite common and desired in the context of high rates of violence against women in South Africa				Measuring both the level of care partner has for safety and heightened surveillance of whereabouts	Revising item to be more specific in order to capture an unhealthy level of surveillance versus general concern for safety or create two distinct items
10. My partner expects me to do everything for him	21% Strongly agreed/Agreed 79% Strongly Disagreed/Disagreed			Item judged as being for married people	“Everything” was vague and many young women asked what was meant by this		Revise item to be more specific. Consider modifying item so that it reflects the common ways in which young women and men in South Africa have relationships
11. Because my partner buys me things, he expects me to please him	5% Strongly agreed/Agreed 95% Strongly Disagreed/Disagreed				Lack of clarity in what was meant by “please him”		Revise item to be more specific Given the lack of agreement to this item, future scales may want to revise this item so it is not interpreted as participating in transactional sex or sex work
12. My partner lets me know that I am not his only girlfriend	15% Strongly agreed/Agreed 85% Strongly Disagreed/Disagreed	This item was often interpreted as ‘does your partner cheat on you’ and participants spoke of finding out their partner cheated through seeing messages on his phone				This item assumes monogamy, and some participants talked about being in an open relationship	Consider removing or modifying in order to ensure the item is more understandable and inclusive of different types of relationships
13. My partner expects me to sleep over whenever he chooses	30% Strongly agreed/Agreed 70% Strongly Disagreed/Disagreed					Item assumes that participants have started sleeping together and are able to have sleep overs	Consider adding some clarity regarding whether participants are able to have sleep overs

**Table 4:** Item appraisal among young men who participated in the cognitive interviews and recommendations for adaptations for the use of the SRPS among South African young men (*n* = 17)

	Response breakdown	Cognitive Process Coding			Question Feature Coding		Recommendations for adaptations
SRPS Scale Item		Comprehension	Response Process	Judgements related to items	Clarity of items (wording, vague)	Logical problems in assumptions (inappropriate assumptions, double-barreled questions)	
1. I am quite comfortable when my partner greets men, she knows	81% Strongly agreed/Agreed 19% Strongly Disagreed/Disagreed			Participants expressed being comfortable with partner greeting men as long as she is not flirting	Lacking clarity in what “greets” was referring to. Not specific enough about which men she is greeting (e.g., friends/family vs. strangers)	Item assumes heterosexuality and that it would be only an issue if participant’s partner was greeting men on the street	Include more specific language so as to avoid confusion
2. I like my partner to be at home when I come to check her, it bothers me if she is not there	69% Strongly agreed/Agreed 31% Strongly Disagreed/Disagreed	Often item was interpreted as participants had already made plans in advance	At times participants responded “it depends” to this question		Young men often assumed this was in the context of them having had made plans with their partner and feeling that they would be upset based on wasting time, thus more context was needed		Clarify whether plans have been made in advance, or consider revising to be more contemporary, understanding that young people are more connected (e.g., telephonically, virtually) than when the scale was originally developed
3. I become jealous when my partner wears things that make her look too beautiful	31% Strongly agreed/Agreed 69% Strongly Disagreed/Disagreed	Some young men were unable to comprehend how someone could get jealous if their partner looked beautiful	Some participants responded “somewhat agree”			Young men sometimes felt that the item was asking both about beauty and how this beauty represented them, which they appreciated but also that wearing revealing clothes was different and would make them jealous	Include more specific language so as to avoid confusion. For example specify whether participant is jealous of partner wearing revealing clothes versus the broad concept of beauty
4. I have more to say than my partner does about important decisions that affect us	60% Strongly agreed/Agreed 40% Strongly Disagreed/Disagreed		Some participants responded “No, she has more.” or “I do”				Responses for this item should consider whether it would be better to understand who in the relationship makes most of the decisions and then providing response options of you, your partner, or both equally
5. I never tell my partner who she can see or spend time with	69% Strongly agreed/Agreed 31% Strongly Disagreed/Disagreed	“Never” was often overlooked in the comprehension of this item					Avoid using negatively worded items that create complicated double negative cognitive processes
6. It might make me sad but my partner is free to leave our relationship any time she wants to	79% Strongly agreed/Agreed 21% Strongly Disagreed/Disagreed	Interpretation was often based on young men’s desire to not breakup more than forcing partner to stay in relationship	Some participants responded they weren’t sure not because they wanted to force their partner to stay in the relationship but because they have tried to breakup and it hasn’t worked				Make item more specific to ensure the scale is capturing control and being forced to stay Future scales may want to consider adding items related to love and building healthy relationships
7. I like to do what I want, even if my partner doesn’t want me to	31% Strongly agreed/Agreed 69% Strongly Disagreed/Disagreed						
8. When my partner and I disagree, I get my way most of the time	31% Strongly agreed/Agreed 69% Strongly Disagreed/Disagreed		Participants often wanted to answer items with yes or no				
9. I like to know where my partner is most of the time	87% Strongly agreed/Agreed 13% Strongly Disagreed/Disagreed	This item was often interpreted as showing care in the context of high rates of violence against women				Both about safety and making sure partner wasn’t with other men	Revising item to be more specific in order to capture an unhealthy level of surveillance versus general concern for safety
10. I expect my partner to do things for me like my ironing and cooking	62% Strongly agreed/Agreed 38% Strongly Disagreed/Disagreed		Participants often wanted to answer items with yes or no	This item was often stated by participants as not applicable because the participant was not married or hadn’t paid lobolo			Specifying whether asking about current situation in young men’s relationship or expectations in the future if they get married/paid lobolo
11. Because I buy my partner things, I expect her to please me	25% Strongly agreed/Agreed 75% Strongly Disagreed/ Disagreed	Some young men interpreted this item as whether they are able to provide for their partners					Future scales may want to consider adding items about young men’s perceived obligation and ability to provide for their partners
12. I let my partner know that she is not the only girlfriend I have or could have	19% Strongly agreed/Agreed 81% Strongly Disagreed/ Disagreed	For some this item seemed implausible (because it would surely end the relationship) Item was interpreted as being honest or that by not telling their partner they have “side chicks” they are protecting her				Assumes that participant is in a monogamous relationship and does not consider potential for open relationships	Item should be revised to better capture whether or not young men are using threats of relationships with other woman as a means to cont
13. When I want my partner to sleep over, I expect her to agree	40% Strongly agreed/Agreed 60% Strongly Disagreed/ Disagreed	Some young men interpreted this item as wanting to spend quality bonding time with their partner and thus having the expectation she will want to sleep over				Item assumes that participants have started sleeping together and are able to have sleep overs	Future scales should consider adding items about expectations for quality time as well as sex with their partners

### Cognitive processes coding

#### Comprehension

Many participants lacked comprehension regarding scale item 5. This was particularly prevalent among young men who seemed to overlook the word ‘never’ in the phrase ‘I never tell my partner who she can spend time with’.

This item was the only item in the scale that was negatively worded. Agreeing to this item would have resulted in greater SRPS scores, as the scale was coded so that higher scores reflected greater SRP equity. While negative items are often placed in scales in order to avoid automatic processing [50], critics of this approach have raised concern about whether or not positively and negatively worded items are measuring the same construct [51]. In our prior work with the SRPS [14], factor analyses among young men found that this item loaded negatively on the one-factor. At the time this seemed counter intuitive, however cognitive interview results highlight how this was likely due to confusion and oversight of the negative wording of this item, especially for young men.

Some items were seen as so implausible in young people’s relationships that participants felt they could not even answer the question. For example, in relation to item; “I let my partner know she is not the only girlfriend I have or could have” one young man from Durban stated:

“Eeeh, it is the one that you asked me that if I cheat on her, will I tell her that. It does not make any sense at all. That is why I couldn’t answer that one.” – Participant 41

Some items were not interpreted as originally intended. For example, although most young men agreed to item 6 “Although it might make me sad, my partner is free to leave the relationship anytime she wants” those that disagreed described wanting to figure out why their partner wants to leave instead of just letting the relationship fall apart. For example, one young man in Soweto stated:

“No, I have not come across that, that one of breaking up, to break up, [no] it would just be conflicts and we would solve them, you see? […] I will not just let her, I need to ask why she is leaving, what happened to [us].” -Participant 48

Similarly, some young women who disagreed to this item also referenced how they wouldn’t (not necessarily that they couldn’t) leave because they loved their partner.

These differences in interpretation by young people highlight the lack of research, particularly in contexts where HIV and gender-based violence is high, focused on how young people perceive and enact love and problem solving within their relationships [52].

#### Judgements related to the items

One common judgement towards scale items raised by young women and young men was regarding how some items were not applicable to their current relationships as they seemed to be for married couples. For example, when responding to item 10 “My partner expects me to do everything for him”, one young woman from Durban stated:

“We are not married people, married people do that. He has not even paid for lobolo, so no. […] No, he must not expect me to…Yo! I have a lot of things to do and now I must leave them and attend to his needs? […] No, ha, never. He is not my husband. I do all of that if I want to.”*-* Participant 165

Even among the 38% who disagreed with this item, discussions of future expectations after paying lobola (bride price) were common.

Finally, several young women discussed how they felt item 5 “my partner never tells me who I can spend time with” was not applicable to their lives because they chose to not have friends and thus their partner telling them who to spend time with was not an issue. Future research should explore the implications and potential consequences of young women having few to no connections with peers outside of their relationship with their partner.

### Question feature coding

#### Clarity of the items (e.g., wording, whether the question is vague)

Numerous items lacked clarity. For example, item one “I am quite comfortable when my partner greets men she knows” raised discussion among some young men regarding the interpretability of the word “comfortable”, with some young men suggesting the item could be worded as “ok” instead of comfortable. For example, one young man in Durban described some of the issues he found with this item:

“Ahhh, okay, it is not that clear because it just mentions “greet”, it does not mention uhm, “talking to”, because greeting and talking to someone is two different things. When she greets someone and does not talk to them, it makes me a bit suspicious. So, it is better if you phrase this question like this rather than saying “talking to” someone because if she talked to someone, she would have an explanation for talking to them, expect for just greeting them, she could make just any excuse and just be like “No, it is a friend”, when it is actually a side person or someone else.” *–* Participant 13

Issues with this item highlight the subtle differences that are important to understand as they could have different interpretations.

Item 2, which for young men stated “I like my partner to be home when I come to check her, it bothers me if she is not there” lacked clarity, which is well explained by one young man from Durban who stated:

“Eeh, this one I, my understanding with it is that, It want to know how I feel when I alerted my partner that I will meet her at her place, and then when I do come and then she is not there, uhhhm then my thought about the question is, yes I agree with the question because I will like my partner to be at her place when I come to check her, because if, because I only come to visit her when I have informed her that on a particular day and time I will come and check you at your place, and then when I eventually do come and then she is not there, then it became problematic for me, because now she is wasting my time, wasted money to travel from my place to her place, only for me not to find her at her place, so that become problematic.” *–* Participant 67

Young women also were confused by this question and would at times ask the interviewer for more context. Thus, while 69% of young men and 50% of young women agreed to this item, the majority agreed because they felt their partner would be upset if they had made plans and then they were not at home when they came to follow through with prior plans. The lack of clarity in this item raises important consideration regarding the ways in which young people communicate through social media and using smart phones and location sharing. This should be considered in more contemporary versions of this scale for use among young people.

For women, item 10 stated; “my partner expects me to do everything for him”, which led to a lack of clarity. For example one young woman from Soweto questioned:

“When you say everything, you mean like house chores eh, laundry, financially?”- Participant 135

Another item which lacked clarity for some young women was item 11; “because your partner buys you things, he expects you to please him”. Participants were at times unclear what was meant by “pleasing him”, suggesting that this and many other items in the SRPS could be revised to be more specific to avoid confusion and improve comprehensiveness.

### Logical problems in assumptions (e.g., inappropriate assumptions, assumptions of constant behaviour, and double-barreled questions)

#### Inappropriate assumptions and assumptions of constant behaviour

Most of the items in the scale were heteronormative and cisnormative, asking young women about their male partners and young men about their female partners. While only one participant described how this raised some issues in responding to the scale, being that she was in a lesbian relationship, future research is needed to understand how power and control functions in non-heterosexual relationships, and how the SRPS could be adapted to better capture diversity in the relationships of young people.

Item 13, which for young woman stated, “my partner expects me to sleep over whenever he chooses”, assumes that young people can have sleep overs, or that they have started having sex. Some of the young women discussed how they hadn’t started having sleep overs or that they aren’t able to have sleep overs because of family dynamics, whereby at least one person in the relationship may still be living with their parents, thus making sleep overs challenging to navigate. For example, one young woman in Durban stated:

“No, […] I live with my parents and I cannot sleep over. […] He knows that my parents are very strict parents and so, he knows that I cannot sleep over whenever he wants me to.” *–* Participant 195

Future studies may want to consider adapting this item to reflect differing family dynamics, abilities for young people to navigate sleepovers while staying at home with their parents, and for sexually active and inactive youth.

#### Double-barreled questions

A few of the items seemed to be capturing multiple important elements in young people’s relationships and thus were left to interpretation. For example, item 3 which for young men stated “I get jealous if my partner wears clothes that make her look too beautiful” was at times understood to be that the men’s partners dressed nice and represented them which they appreciated, while some young men interpreted this item to mean that their partner wore revealing clothing and this brought attention from other men, which led to jealousy. One young man from Durban expressed:

“So, in my understanding, if she wears something that make her look beautiful, I am okay with it, but if she wears something that exposes her. I am not quite happy with that. So, I answered this question based on my understanding of beautiful rather than the society’s.” -Participant 13

Discussions raised from this item brought insight into different interpretations of beauty and the role and importance of gendered beauty standards, and how young women must navigate the fine line between being beautiful and attractive to their partners while at the same time ensuring they aren’t dressing too provocatively as to upset them. For example, one young woman from Soweto describes her interpretations of what beautiful means to her in the context of the scale and what the item is trying to measure:

“Not really, ehm, I guess it’s something it’s an issue that I always had like from growing up, I never liked short things [revealing clothes], so I feel like they are uncomfortable, so that’s why I always avoid wearing them, like if you appear wearing short things it can mean a lot of things, like putting a lot of makeup, your weaves on like, from being simple, from having like a natural hair to relaxing your hair which will make you maybe more beautiful or wearing wigs, so ja, no, but in this question mostly, I would say maybe it’s wearing short, for me, it’s wearing short [clothing] ‘cause I don’t apply as well a lot of make-up.” *–* Participant 11

For this young woman, even though the issue of her partner being jealous if she looks “too beautiful” was not relevant in her relationship, it clearly shows how there are multiple societal pressures for young Black women in South Africa to look and dress a certain way to be perceived as beautiful. The importance of beauty and attractiveness has not been widely investigated within the relationships of young people, thus future research is needed to explore the role of beauty and beauty standards in the relationships of young South Africans.

Young women also raised concerns around multiple interpretations of item 7 “My partner does what he wants even if I don’t want him to”. For example, one young woman from Soweto stated:

“Are you saying that’s what he wants, in which sense, like him maybe going out to watch soccer or is it when I say, I don’t want you to touch me when he touches me or do you mean?” *–* Participant 116

This highlights the potential dual interpretation of this item that could be about one’s partner going out and doing things that you don’t want them to do, or that they are making unwanted sexual advances or perpetrating sexual violence. These are two distinct and important relationship issues, that future scales may wish to measure as separate items.

Finally, as highlighted in the title of this paper, both young men and women felt that item 9, which for young women stated, “My partner always wants to know where I am”, captured both elements of caring and over-surveying or controlling behaviour. One young woman from Durban captured this issue with double-barreled interpretations when she stated:

“Uhm…Jah, I feel like there is a twist somewhere, somehow […] I feel like it is asking if he is a stalker… […] But I also feel like it is asking if he cares.”- Participant 129

Young men from both sites also discussed how in the context of South Africa where there are high rates of violence this item could be interpreted as caring and trying to protect partner from violence. For example, one young man from Soweto stated:

“If I know where she is and I am not with her, I become so free to say, okay, my partner is at a certain place, she is doing 1, 2, 3, even though I don’t see her […] but I have peace that I mustn’t worry too much about her. If she does not tell me where she went, I will be worried, worse, if I call and she does not take my call, then I will think that eish since these days, there is human trafficking.” -Participant 49

In South Africa, a woman is killed every 3 hours, and femicide rates are five times higher than the global average [53]. Under this backdrop, it is no surprise that 65% of young women and 87% of young men agreed to this item, which although originally aimed to capture surveillance and controlling behaviour by male partners in heterosexual relationships, was interpreted by many young women and men, not as sign of control, but one of care, concern, and protection.

## Suggestions for revisions and adaptions

Young women and men had lots of suggestions for how to improve the scale, including advice on being more specific, rewording items, adding additional questions about power and control, including questions that were more general about relationship dynamics, as well as adding questions about health, sexual behaviour, and violence and abuse.

To address some of the issues raised by participants about items in the SRPS, participants brought up suggestions for making items more specific. For example, one young woman from Soweto stated:

“Maybe if we can just…, when you ask the question, maybe add more details so that I know that when I respond I will give you the answer that is appropriate ‘cause now it’s open-ended you know I can say yes, I agree with just everything but then you find that another person perceives it differently, so ja*” –* Participant 11

Another young woman in Soweto provided specific recommendations for item 1 “My partner is comfortable when I greet men I know”, stating:

“I think that’s how it should be, like more specific whether in public or in private space.”-Participant 111

Also, given that the scale was asked about young women’s partner’s behaviour, some young women suggested that items be added to also assess their own behaviours.

Both young women and men brought up several suggestions for items that they felt would be important to include to measure power and control in relationships. This included asking participants if they believe in gender equality/equity, if you go out with friends, and if you allow your partner to have a say about decisions in the relationship. For example, one young man from Durban stated:

“I will speak in a manner we usually speak with the guys here in Durban, I will say hey my brother how do you feel about the 50/50 [gender equality] thing?”- Participant 41

This also highlights how some of the items could be reworded to better reflect the ways in which young people talk about SRP in South Africa.

Specific questions about relationship dynamics were of interest to participants including whether your partner takes you on dates, questions about intimacy, and plans to have children together in the future, as well as emotional wellbeing in your relationship. When asked about SRP in South African youths’ relationships, one young woman from Durban stated:

*“*The emotional wellbeing of a person in that relationship. How are you fitting in emotionally? Because some people can be like. Yes, he understands me but there is that emotional part of them where they are breaking. Where they are not happy emotionally. But in other things they can defend their partner and say yesss he is a good person but emotionally the soul is the important thing,” *–* Participant 123

These suggestions call for increased attention to strength-based measures of gender equity that focus on positive assets of relationships and deeper connections that young people have with each other.

This desire to explore in greater detail the intricacies of young people’s relationships came up through suggestions to include additional details of young people’s sexual relationships, as a marker for relationship satisfactions. For example, one young man from Durban suggested:

“Like how, how often do you have sex with your partner or how much sex should one have with her partner per month or per week or, ya, those kinds of questions. […] Well, it also depends on how active, how sexually active you are. If you are very sexually active and your partner is not around, the chances are high that you will find sexual pleasure from someone else, other than your partner.” – Participant 67

Several suggestions came up around sexual health and sexual violence and abuse such as whether you would ever force your partner to have sex with you, if your partner was abusing you, and asking about who in the relationship might have more physical power. Concerns about cheating and the consequences of cheating were also discussed as important for understanding power dynamics in young people’s relationships. This included suggestions to ask questions about whether participants ever got an “infection” (STI or HIV) from a partner and what they did about it. Other questions about health were suggested including asking whether participants ever went to get tested for HIV with their partner.

Questions about employment and how economic inequities and societal gender roles may impact relationships were also suggested by participants. For example, one young man from Durban stated:

“Maybe what can it be, ooh maybe it can be a job. Maybe if someone have a better job, does that affect the relationship. If I as a male work better and earn better, would it make me not to respect my partner during that period.” *–* Participant 58

This recommendation highlights the intersecting nature of sexual relationship power inequities and ‘gender role strain/stress’ or men’s stress related to inability to achieve hegemonic forms of masculinity including the ability to provide for one’s family through work [54–56]. This construct has been measured and explored among South African men using the gender role strain scale, to better understand how multiple forms of masculine identities are formed in response to gender role strain, and how in turn these identities and beliefs about gender roles influence important sexual and relationship behaviours and outcomes [57–59]. While quantitative validity evidence has been established among young men in South Africa, future research should consider how both young men and young women perceive existing measures of gender role strain [59].

Finally, both young men and women spoke about adding questions about technology and looking at each other’s phones in relationships.

## Discussion, recommendation, and reflections

The results from this study highlight multiple issues surrounding items that have been used in youth sexual and reproductive health studies in South Africa. Given that the SRPS was originally developed among women in the US in the year 2000, and the inherently dynamic nature of gender roles, dynamics, and identities [60], it is not overly surprising that many of the findings highlighted how the scale may not be contemporary for youth in present-day South Africa. This included that items did not acknowledge the current ways in which youth communicate, particularly when it comes to technology, cellphone use, and social media. Another key finding from this study was that the scale is extremely heteronormative and the wording of most of the items were problematic for youth who were in non-heterosexual or open relationships. The lack of applicability of scale items was also discussed in conversations surrounding youth’s perceptions that many of the scale items seemed to be for married people. Scale items were also limited in their ability to measure equitable relationship dynamics, including intimacy and love. Given these issues, there are many recommendations made by participants that could help to make the scale more contemporary and relevant for understanding power dynamics within South African youth’s relationships.

The use of technology and communication through social media is an important area for future research. For young people in today’s society, relationship formation, sex, and love most often begins and is sustained online and through social media and other dating apps [61]. Thus, the results from this study further highlight the critical need for measures, such as the SRPS, to be adapted to reflect the increasingly virtual ways in which young people form and perform relationships. Further investigation into different modalities that youth use to communicate with each other and seek supports for relationship challenges is warranted, given that mobile technology infrastructure is highly developed and used by populations of youth people in South Africa, who may otherwise have limited available options to discuss challenging issues related to gender inequities [62–65].

Many participants brought up that the items in the scale seemed to be for married couples and weren’t relevant, as most participants were unmarried. Previous research has highlighted that even young men who are not yet married have perceived entitlement to any woman to whom they will have to pay lobola to marry [66]. This is concerning as previous research has found that this entitlement can lead to increased demands for unsafe sex in relationships [67]. In the context of South Africa, where many young people are not married, or do not get married until later in life [68], items that focused on the importance of decision-making, household duties, and breakups may be less relevant or thought of differently for young people who are in more casual relationships or not cohabitating with their partners. For example, youth in more casual non-cohabitating relationships may feel that it is easier to leave relationships or breakup with their partner over the phone than youth in more serious cohabitating relationships. As such, measures should consider the seriousness of youth’s relationships and distinguish between current expectations and future relationship expectations.

In contexts like South Africa, where HIV is endemic, youth relationships are rarely discussed through a strength-based lens, instead researchers and program and policy makers have mostly focused on deficit-based narratives of youth sexual relationships as inherently risky [69]. While there is increased attention to strength-based research and measurement development with Indigenous communities globally [70], and in the field of mental health [71], strength-based measures for gender equity and SRH are not widely available. Many of the discussions and recommendations made by young women and men in our study centered on the lack of focus on positive sexuality. This included recommendations for the addition of items which ask about partner communication regarding sexual health, HIV testing, and sexual behaviour more generally. Partner notification is an important aspect of sexual health and STI/HIV prevention and control, especially in contexts like South Africa where STI care is done through syndromic management [72]. Power and control influence the ability and level of comfort in disclosing HIV and STI testing and status, thus scales should include items that explore young people’s ability to get tested with their partners, as well as items exploring young people’s comfort and preference for STI and HIV disclosure in relationships.

Findings also highlighted how partners as well as parents influenced youths’ relationship dynamics. The items in the SRPS stem from the Northern assumption that decision-making happens at the individual-level, whereas in South Africa, and many other global settings, decision-making is largely relational [73]. Thus, recommendations to include or adapt items to acknowledge the relational importance and influence on SRP equity and sexual decision-making, is in line with calls for the adoption of relational theory as a framework for understanding gender and health issues on a global scale [7]. Moreover, measures and efforts need to move away from deficit-oriented narratives to better understand the ways in which programs and policies can be implemented to build healthier relationships and more positive and open conversations about sexuality between young women and men, as well as with their families, as a pathway to achieve gender equity, SRH outcomes, and the overall wellbeing of young South African women and men across the life course.

Issues raised by participants were not equal across items or genders. Overall, data indicated that young men raised more comprehension issues with the scale items, which is not overly surprising given that the scale was originally developed to measure women’s perceptions of their male partners controlling behaviours and dominance in relationships [3]. While there were issues raised across all items among women, among men the item “I like to do what I want, even if my partner doesn’t want me to” did not raise any specific issues. Issues relating to response processes only did not seem to affect the overall interpretation and ability to measure SRP equity. For example, item 8 “When my partner and I disagree, he gets his way most of the time” both young women and men often stated ‘it depends’ or used a yes or no response, but the item itself was overall well understood. Whereas issues in double barreled interpretations and comprehension affected the validity of the item, resulting in some items not accurately capturing sexual relationship power dynamics. For example, young women and men interpreted item 9 “I like to know where my partner is most of the time” most often as a caring and positive behaviour and not as a sign of control or dominance.

Future research wishing to use the SRPS in their research with youth globally should consider the recommendations and suggestions for adapting the scale as mentioned by the participants and outlined in Tables 3 and 4:

In general, some recommendations for revising the scale include:

1. Revising the scale language to be more inclusive of gender and sexual diversity as well as different types of relationships (e.g., open relationships or non-cohabitating relationships that may involve children).
2. Modifying items so that they have contemporary relevance. This could include ensuring translations of the scale match the age and group context. Also, to reflect youth’s realities, we recommend modifying items about one another’s whereabouts to capture how young people are more connected via technology than they were when the scale was developed. We also recommend revising items that may not make sense for youth in South Africa who generally aren’t married or living with their partners.
3. Adding or modifying items so that they reflect the multidimensional and dynamic aspects of gender and SRP equity, including the importance of relationships at the intimate partner, peer, community, institution, and societal levels.
4. Revising items to be more strength-based, to accurately capture equitable relationship power dynamics and shift the focus away from deficit-based narratives of inequity and risky youth sexuality.
5. Revising some of the items to be more specific to avoid differing and multiple interpretations
6. Exploring whether different response options would make more sense for certain items. Also, if response items should have the option for participants to respond don’t know/unsure. For example, providing the options of: Always, often, sometimes, rarely, and never. This could also include exploring how having more than 4 response options and having a neutral option might affect the responses and overall scale scores.

These recommendations highlight the complexity of creating a one-for-all measure for complex constructs such as SRP equity. Moreover, the ever-changing and involving nature of gendered power dynamics will require researchers to continuously evaluate, modify, and adapt measures to reflect these changes, further complicating efforts to monitor progress towards global goals for gender equality [7]. However, efforts grounded in community-based approaches and theories, theoretical frameworks, and evidence produced by and with scholars in the Global South can support the development and greater use of measures that reflect the living realities of communities in the Global South. Together, these efforts support the potential for better global monitoring of key determinants of health and in turn the advancement of gender and health equity for all.

### Strengths and limitations

Participants for this qualitative study were recruited from a larger sample of youth who participated in a longitudinal study ‘AYAZAZI’ from 2015 to 2017. Participants, however, had not been in contact with the study for several years. While many participants were unreachable, there were no major differences between participants who were and were not ﻿﻿included in this sub-study (Additional file 2: Supplementary Table 1). A moderate number of participants were still able to be contacted years after their original participation. Providing youth friendly spaces, training research staff to be youth allies, and allowing youth to discuss important issues in their lives makes an impact and allows for greater opportunity to reconnect with young people for follow-up research and engagement.

Success in recontacting participants several years following the end of the AYAZAZI study is likely attributable to the youth engagement approach undertaken by the study, and high retention rates during the cohort follow-up, which was facilitated by ongoing youth-friendly efforts to reconnect with participants. These included knowledge translation and exchange events, follow-up studies, and social media engagement. Recruitment findings highlight important gendered considerations for recontacting youth in follow-up research. Overall, it was more challenging to reach young men. In Durban, young women were more likely to decline participation. While in Soweto, where all interviews were conducted telephonically, young men, once reached, were more likely to decline to participate or were unreachable after agreeing to participate. The change in the interviews’ modality from in-person to over the phone due to the COVID-19 pandemic may have also influenced study participation differently for young women and men. For example, every contacted woman in Soweto agreed to participate in CIs versus only 23% of young women in Durban. This provides potential indication that young women may have felt more comfortable participating in research over the phone, especially research discussing challenging relationship issues. Whereas young men seemed willing and agreeable to participate in research, however other competing demands, or feeling of discomfort with discussing relationship dynamics over the phone, may have kept them from participating at the scheduled time.

Our results found that since the 12-month AYAZAZI questionnaire was completed, young women had greater SRPS scores, and young men had lower SRPS scores. Differences across study visits were unlikely to be because of the sample recruited. When comparing responses among the participants who answered the SRPS scale at the 12-month AYAZAZI questionnaire and who participated in the CIs, the effects of the change were greater (Table 2). Thus, differences in scores may have been due to changes in relationships, a function of older age, shifts in gender norms by participants, or changes in the interpretation of the scale items over time. Issues with measurement invariance as participants age and mature has been recently discussed using the gender equity men’s scale in a cohort of young women in South Africa [74]. Other issues affecting the scores could have been due to the response options provided, whereby for several items participants did not respond with the given response options of strongly agree to strongly disagree. Instead, some participants responded with yes or no answers, or other responses (e.g., sometimes, it depends). Tables 3 and 4 presents additional response option issues for all applicable items. Future studies utilizing and adapting the SRPS for use among youth may want to explore different response options to the items including Likert-Type Scales that range from always to never instead of strongly agree to strongly disagree.

Improvements in SRP among young women in our study is different than prior research among young women in Kenya, highlighting reductions in SRP with age [49]. This, however, could be partially explained by the fact that this study only included young women up to the age of 24, and did not look at changes in SRP among the same women over time. While no study to our knowledge has examined changes in SRP equity overtime among young women or men in sub-Saharan African contexts, a study among young women and men in Uganda found that younger adolescents (aged 10–14) had more gender inequitable beliefs than their older peers, highlighting that young people may become more gender equitable, or at least report more gender equitable attitudes, beliefs, and behaviours, with age [75]. While this change was not statistically significant, given our small sample (*n* = 17), may still have meaningful implications for shifts in young men’s tendency to exert greater control and display adult dominant masculinity, as they get older [76]. There is, however, a paucity of literature exploring how gender equitable beliefs, norms, and behaviours shift with age, thus requiring further research [74].

The use of cognitive interviewing in our study provided an opportunity to establish a youth-engaged process for appraising items of a well-established measure of gender equity that provided insight, evidence, and recommendations for scale adaptation strategies for the use of the SRPS in South African youth studies, and specifically how the SRPS may function differently for young women and men. However, due to limitations in recontacting participants several years after last contact with our study team, we were unable to fully follow the intended purposeful sampling approach. Our data collection took place over a period of 15 months due to the ongoing COVID-19 pandemic causing interruptions in data collection and requiring the study team to re-submit ethical approval to continue interviews telephonically. Interviews that were conducted telephonically may have differed from those done in-person pre-COVID, however the interviewers felt that they were able to have meaningful conversations with participants despite not being face-to-face. In fact, the youth interviewer in Soweto (MK), interviewing young women, felt that young women may have been able to be more open about their relationships than would have been possible in person. Telephonic interviews also allowed participants who may have moved out of the area to participate, and thus may have been why all the young women who were contacted in Soweto participated in the cognitive interviews. Moreover, our results present a disproportionate number of quotes from young men in Durban, which was reflective of young men residing in Durban, the majority of whom completed the CI in person, having more to say about the items in the scales than young men in Soweto, all of whom conducted the survey telephonically. These findings raise important gendered considerations for data collection and the importance of exploring youth preference for data collection methods and modalities (e.g., in person vs. mobile/telephonic) in future youth studies focused on topics related to sexuality, relationships, and gender norms and roles.

While detailed socio-demographic information (e.g., socio-economic status (SES), education, food-security [77]), mental health (e.g., stress [78], depression [64]), experiences of violence [14], and substance use were captured during the quantitative AYAZAZI questionnaire, we did not re-ask participants about these factors at the time of the qualitative cognitive interview (3–5 years later), as such we were limited in the ability to compare findings across these factors.

Finally, our team spent an extensive amount of time reviewing the data, co-coding, and double coding transcripts; however, this cannot prevent our own world views and values from impacting the interpretation of the data. Because members of the team spoke different languages, and interviews were conducted in multiple languages, they had to be translated into English in order to be discussed as a group, which may have posed issues in cross-cultural interpretation of the data [34]. As this project progresses, we intend to continuously share and incorporate the views of multiple audiences into future findings and will continue to support youth capacity building in our research program.

## Conclusions

Given the wide use of the SRPS in research centered on youth’s SRH, this study fills an important gap in unpacking the validity evidence of the scale and provides insights into the gendered comprehensiveness and contemporary relevance of the SRPS in the lives and relationships of young women and men in South Africa. While many of the participants felt that the scale adequately captured SRP and were relevant to their relationships, and the relationships of their peers, this differed by gender, and there were several items which were interpreted differently than the original scale intended. Moreover, even when reminded of the response options, many participants chose to answer the items using their own responses, raising potential issues in validity evidence surrounding scale content as well as response consistencies. Numerous recommendations for additional and more contemporary, relational, and relevant measures of sexual relationship power were suggested by participants, providing opportunity for researchers to adapt youth recommendations into future use of the SRPS in their research and program evaluations *by, with, and for* youth. To address gender equity and improve the health and well-being of youth across the life course, the validity evidence of measures used within youth research needs to be evaluated on an ongoing basis to ensure measures remain contemporary and reflect the living realities of young people. As such, the methods used in this study could be applied across a range of disciplines and settings to support the meaningful engagement and participation of affected communities to improve measurement development, adaptation, and appraisal.

## Supplementary Information

**Additional file 1: Supplementary material.** Details of the Cognitive Interview training.

**Additional file 2: Supplementary table 1.** Comparing participants in Cognitive Interview study to the rest of the AYAZAZI sample.

## Data Availability

The Cognitive Interview data is not available for sharing, as we did not receive consent to share or archive transcripts from the participants. Please feel free to contact Kalysha Closson if there are any inquiries regarding the data.
