# Supplementary material for: “I feel like it is asking if he is a stalker … but I also feel like it is asking if he cares”: exploring young South African women and men’s perceptions of the Sexual Relationship Power Scale
Source: BMC Public Health. 2022 Jul 16;22:1368. doi: 10.1186/s12889-022-13686-9 (PMC9288208; doi:10.1186/s12889-022-13686-9)
Supplement: Supplementary file 1 — Additional file 1: Supplementary material. Details of the Cognitive Interview training. [file 12889_2022_13686_MOESM1_ESM.docx]

Supplementary material- Details of the Cognitive Interview training

The virtual cognitive interview training consisted of five virtual meetings with two interviewers:

- 1. Informal meeting to introduce team members and overview of the project.
  2. Formal presentation detailing the study background, purpose of the study, overview of cognitive interviewing and validity broadly, practicing think aloud procedure and probing, discussing taking notes and detailing body language and providing interview summaries.
  3. Training from local expert in qualitative research Mamakiri Mulaudzi, PhD Candidate and Senior Researcher at the Perinatal HIV Research Unit. Qualitative research training provided an overview of why and how qualitative data is collected including different types of interviewing, how to choose a sample size, how to create an interview guide, dos and don’ts of interviewing, and multiple role-plays to practice managing difficult situations during the interview. Interviewers were also provided a checklist for conducting in-depth interviews (See **Supplementary Material**).
  4. Online workshop on cognitive interviewing hosted by the University of Cape Town School of Public Health which introduced participants to cognitive interviewing and provided an overview of methodological considerations (1).
  5. Second training presentation and discussing script for contacting participants, call log, and interview tips from one of the interviewers who conducted interviews in Durban

In between training meetings, interviewers met to practice mock interviews with one another. Interviewers were trained to use flexible interview techniques and probing to further inquire about areas of interest as they emerged. Interviewers were trained to specifically ask the survey item word-for word and to avoid explaining if the respondent was uncertain what the question was asking.

1. Scott K, LeFevre AE, Ummer O. An introduction to cognitive interviewing: How can we enhance survey questionnaires to ensure that they actually measure what we want to know? In: University of Cape Town School of Public Health, editor. 2020.

2. Ballard T, Coates J, Swindale A, Deitchler M. Household Hunger Scale:

Indicator Definition and Measurement Guide. Washington, DC: Food and Nutrition Technical

Assistance II Project; 2011.

3. Radloff LS. The CES-D scale a self-report depression scale for research in the general population. Applied Psychological Measurement 1977;1(3):385-401.
