# Supplementary material for: “I feel like it is asking if he is a stalker … but I also feel like it is asking if he cares”: exploring young South African women and men’s perceptions of the Sexual Relationship Power Scale
Source: BMC Public Health. 2022 Jul 16;22:1368. doi: 10.1186/s12889-022-13686-9 (PMC9288208; doi:10.1186/s12889-022-13686-9)
Supplement: Supplementary file 2 — Additional file 2: Supplementary table 1. Comparing participants in Cognitive Interview study to the rest of the AYAZAZI sample. [file 12889_2022_13686_MOESM2_ESM.docx]

Supplementary Table 1– Comparing participants in Cognitive Interview study to the rest of the AYAZAZI sample

|  | Cognitive Interview Participant (n=38) | Non-Cognitive Interview participants (n=354) | P-value |
| --- | --- | --- | --- |
| Site |  |  | 0.982 |
| Soweto | 18 (47.4) | 167 (47.2) |  |
| Durban | 20 (52.6) | 187 (52.8) |  |
| Sexual Orientation |  |  | 0.854 |
| Heterosexual | 35 (92.1) | 328 (92.9) |  |
| Lesbian, Gay, or Bisexual | 3 (7.9) | 25 (7.1) |  |
| Ever had children |  |  | 0.630 |
| No | 30 (79.0) | 267 (75.4) |  |
| Yes | 8 (21.0) | 87 (24.6) |  |
| Number of dependent |  |  | 0.524 |
| 0 | 30 (79.0) | 255 (72.0) |  |
| 1 | 4 (10.5) | 63 (17.8) |  |
| ≥2 | 4 (10.5) | 36 (10.2) |  |
| Language |  |  | **0.042** |
| IsiZulu | 23 (60.5) | 268 (75.7) |  |
| Other | 15 (39.5) | 86 (24.3) |  |
| Any household hunger |  |  | 0.164 |
| No | 37 (97.4) | 321 (90.7) |  |
| Yes | 1 (2.6) | 33 (9.3) |  |
| Any history of drug use (excluding marijuana) |  |  |  |
| No | 36 (94.7) | 332 (93.8) |  |
| Yes | 2 (5.3) | 22 (6.2) |  |
| Formal Housing |  |  | 0.637 |
| Informal | 4 (10.5) | 42 (11.9) |  |
| Formal | 28 (73.7) | 235 (66.4) |  |
| RDP housing | 6 (15.8) | 77 (21.8) |  |
| Ever had sex |  |  | 0.271 |
| No | 7 (18.4) | 43 (12.2) |  |
| Yes | 31 (81.6) | 311 (87.8) |  |
| Had sex with two or more partners in the last 6 months (n=309) |  |  | 0.747 |
| No | 15 (48.4) | 143 (51.4) |  |
| Yes | 16 (51.6) | 135 (48.6) |  |
| Ever experienced physical intimate partner violence? (women only) |  |  | 0.941 |
| No | 17 (81.0) | 175 (80.3) |  |
| Yes | 4 (19.1) | 43 (19.7) |  |
| Probable Depression |  |  | 0.316 |
| No | 13 (34.2) | 151 (42.7) |  |
| Yes | 25 (65.8) | 203 (57.3) |  |

Household hunger measured using the Household Hunger Scale (HSS) (2) (possible range 0-6, with higher scores indicting greater household hunger). A score of ≥1 was considered any household hunger.

Probable **depression** measured using a validated 10-item adaptation of the Radloff, 1977 original 20-item Centre for Epidemiology Depression (CES-D) scale (3) (possible range 0-25, with higher scores indicating more depression symptoms). A score of ≥10 was considered consistent with ‘probable depression’.
